# The contribution of respiratory and hearing protection use to psychological distress in the workplace: a scoping review

**DOI:** 10.1007/s00420-022-01863-7

**Published:** 2022-04-26

**Authors:** Richard Leung, Margaret M. Cook, Mike F. Capra, Kelly R. Johnstone

**Affiliations:** School of Earth and Environmental Sciences, The University of Queensland, Brisbane, Australia

**Keywords:** Mental health, Stress, Masks, Respirators, Hearing protection

## Abstract

**Objective:**

Workers from various industries use personal protective equipment (PPE) including masks, respirators, and hearing protection to reduce their exposures to workplace hazards. Many studies have evaluated the physiological impacts of PPE use, but few have assessed the psychological impacts. The aim of the present study was to carry out a scoping review to compile existing evidence and determine the extent of knowledge on workplace mask, respirator or hearing protection use as a psychosocial hazard (stressor) that could result in a stress response and potentially lead to psychological injury.

**Methods:**

The scoping review followed recognized methods and was conducted using Ovid Emcare, PubMed, Sage Journals, ScienceDirect, Scopus, SpringerLink, Google Scholar and preprint databases (OSF Preprints and medRxiv). Articles on the stressors associated with the use of masks, respirators, and hearing protection were included. The extracted data included author(s) name, year of publication, title of article, study design, population data, stressors assessed, and key findings.

**Results:**

We retrieved 650 articles after removal of duplicates, of which 26 were deemed eligible for inclusion for review. Identified factors associated with PPE use that could potentially create a stress response were identified: communication impacts, physical impacts, psychological illness symptoms, cognitive impacts, and perceived PPE-related impacts. Evidence for respirators suggest that there may be psychological injury associated with their use. However, hearing protection appears to have a protective effect in reducing psychological symptoms such as anxiety, depression, and aggression.

**Conclusions:**

Mask or respirator use may lead to an increase in work-related stress. Whereas hearing protection may have protective effects against psychological symptoms and improves speech intelligibility. More research is needed to better understand potential psychosocial impacts of mask, respirator and/or hearing protection use.

**Supplementary Information:**

The online version contains supplementary material available at 10.1007/s00420-022-01863-7.

## Introduction

Personal protective equipment (PPE) is used by workers in a wide range of industries to reduce exposures to health and safety hazards (Balkhyour et al. 2019). Yet the use of PPE is recognized as the last step in the hierarchy of control to mitigate occupational hazards and is the least effective measure of controlling the risk (Olaru et al. 2021). The hierarchy progresses from elimination of the hazard, through substitution, engineering, administrative measures, and down to PPE usage. Although PPE has its limitations, its use is still considered an important protective barrier when other higher order control methods have been considered and exposure to the hazard cannot be adequately reduced by other means or in emergency response scenarios.

While the intention behind the use of PPE is to protect the worker from potential workplace hazards that could lead to physical harm, there are various physiological effects associated with PPE use that could impact upon a worker’s physical wellbeing. For instance, PPE used on the face or head, specifically hearing protection, surgical masks, and respirators, are accompanied by various mild physical and physiological impacts including pressure-related skin lesions and dermatitis (Battista et al. 2021), perceived heat stress (Scarano et al. 2020), and increased heart rate, respiratory rate, and blood pressure (Lässing et al. 2020).

PPE used on the face (masks and respirators) has been shown to impact on social interaction by creating difficulties in the recognition of faces (Freud et al. 2020) and emotions (Carbon, 2020; Grundmann et al. 2021). The use of this type of PPE can cause fatigue (Wu and McGoogan 2020), which has been shown to negatively impact performance and cognitive function (Möckel et al. 2015; Slimani et al. 2018). It can also impact on communication through reduced speech intelligibility (Randazzo, Koenig, and Priefer), which perceivably could lead to miscommunication within the workplace. Miscommunication can not only interrupt workflow and lower work efficiency but can also lead to misunderstandings and workplace conflicts. Studies have also reported on the impact of mask use, psychological impact, and communication issues in individuals with normal hearing. Malzanni et al. (2021), for instance, reported an overall decrease in quality of life for those who used face masks; specifically, impacting on individuals’ ability to perform in some physical activities and interfering with normal social activities. It was also reported that face masks were the main contributors towards communication difficulties due to sound attenuation and impairment of facial expression recognition. These effects on social interaction and cognition are psychosocial hazards that may impact on occupational stress, which could lead to psychological injury.

Psychological symptoms, such as anxiety and depression, have a bidirectional relationship with stress whereby the symptoms can be both a cause and effect of stress (Daviu et al. 2019; Kinser and Lyon 2014). Normally, the brain regulates how the body responds to stressors to maintain homeostasis of neurotransmission, endocrine levels, and immune function, alongside sympathetic and parasympathetic activity. A balance in these bodily functions contribute towards maintenance of allostasis or psychological and physical balance (Kinser and Lyon 2014; McEwen and Lasley 2003). Short term exposures to stressors can trigger regulatory functions that can enhance an individual’s response to stress and manage negative physiological effects (Epel 2009). However, problems begin to arise when one experiences long-term exposure to stressors unabated which can result in the impairment of neuronal function and regulatory systems (Kinser and Lyon 2014). Chronic exposure to stressors can eventually cause psychological injury and other comorbidities when one’s ability to cope cannot keep up with the repeated episodic stressors (McEwen and Lasley 2003).

Within the workplace, workers are exposed to a wide range of psychosocial hazards which can lead to occupational stress. Stress has both a physical (objective) and psychological (subjective) component (Mariotti 2015). Occupational stress or work-related stress occurs when the resources of an individual are not sufficient enough to cope with the demands of the situation (Basu et al. 2017). How individuals respond to stress largely results from how they anticipate and control a certain stressor (Koolhaas et al. 2011). Studies have linked adverse health and occupational outcomes with stress. Acute exposure to stress can result in gastrointestinal upset, fatigue and disturbance in sleep (Beswick et al. 2006; O’Connor et al. 2021); and those who experience chronic stress exposure are at a higher risk of developing hypertension, cardiovascular disease, psychological injury, and musculoskeletal illness (Basu et al. 2017).

**Fig. 1:**
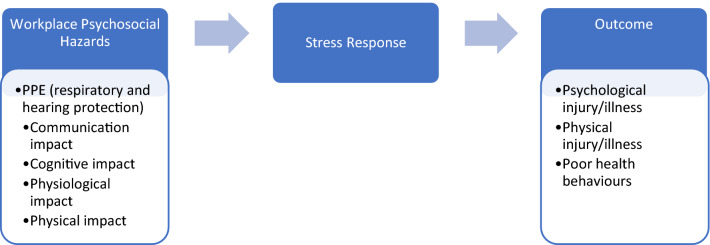
Relationship between workplace psychosocial hazards, stress response, and health outcomes

Figure 1 highlights the relationship between workplace psychosocial hazards such as PPE use, stress response and psychological injury. PPE-related psychosocial hazards/stressors include communication, cognitive, physiological, and physical effects. These PPE-related stressors can then elicit a stress response, to which an individual can overcome the episode or experience psychological distress. If one does not have the resources to cope with repeated stressful episodes, they are at an increased risk of developing psychological injury or illness, physical injury or illness, and poor health behaviors.

The recent coronavirus disease 2019 (COVID-19) pandemic has resulted in an increase in the use of PPE for both the public and in various workplace settings. This has resulted in surgical mask wearing becoming a common occurrence throughout the world. Literature on the various impacts of COVID-19 continues to proliferate with many reporting the influence of mask wearing on psychological health, primarily among health care workers and the public (Amin et al. 2020; Choi et al. 2020). Outside of COVID-19 related research, most research on PPE use, to date, has tended to focus on the physiological impacts and not so much on psychological impacts.

Masks and respirators are used in other industries outside of healthcare and it is anticipated that similar psychological impacts from mask or respirator use may be experienced in these other industries. There is potential for any PPE used on the face or head that may impact on social interaction and cognition (e.g., hearing protection) to act as a psychosocial hazard or stressor. Hence, the purpose of this scoping review is to compile existing evidence on this emerging issue of workplace PPE-related psychological impacts. For the purposes of this review, PPE that will be examined will include those that occlude parts of the face (i.e., nose and mouth) and those that affect the sense of hearing. Specifically, respiratory protection (surgical face masks, and various types of respirators) and hearing protection (earmuffs and earplugs). While there are other forms of PPE that may also create a stress response in the wearer and impact on their psychosocial health (e.g., gloves, gowns, visors, and head protection), this review will focus on PPE that would most likely be used broadly in a wide range of industries.

## Methods

### Study design

A scoping review aims to compile an overview of existing evidence in an area of interest and provide an opportunity to identify key concepts, gaps in the research, and types and sources of evidence to inform practice, policymaking, and guide further research (Arksey and O’Malley 2005; Munn et al. 2018; Pham et al. 2014) Due to the broad nature of personal protective equipment available, the various situations in which they are used, and the lack of any published comprehensive reviews on the topic of PPE use creating a psychosocial hazard, a scoping review was deemed most appropriate for this study. The scoping review was conducted and findings were presented in accordance with the framework outlined by Arksey and O’Malley (2005). The five stages of the framework described were (1) the identification of a clear research question, (2) identification of relevant studies, (3) selection of studies, (4) charting of data, and (5) to collate, summarize, and report the results. The primary research question guiding this review is “what is the extent and scope of research on workplace use of masks, respirators or hearing protection acting as a psychosocial hazard?”.

### Literature search strategies

Relevant peer-reviewed articles were searched using electronic databases and search engines including PubMed, Scopus, Ovid Emcare, SAGE Journals, ScienceDirect, and SpringerLink. These were chosen because during initial searches, the team noticed that some articles were not indexed or available in some databases or search engines. To find further relevant studies that may not have been identified through the databases, we hand searched preprint servers (OSF Preprints and medRxiv), Google Scholar and review articles. The research team limited the search to the past fifteen years [2006 to 2021]. This time period was chosen as it was believed that there would be limited research on this topic outside of healthcare and the extended time period could aid in identifying relevant research. The search strategy involved the use of the Boolean operators “AND” and “OR” when searching keywords. Furthermore, depending on the database being used, Medical Subject Headings (MeSH) were used. The keywords used were terms relating to PPE used on the face and ears (e.g., mask, hearing protection device) and psychological health (e.g., psychological distress, depression, anxiety). The keywords used were searched in the titles, abstract, and text of relevant articles. The keywords used in each search from the chosen databases and search results can be found in Supplementary Table 1. The search was conducted between 5th July and 4th of August, 2021.

### Eligibility criteria

The scoping review included both qualitative and quantitative studies that had assessed the psychological health impact of PPE worn on the face and ears in the workplace, and documents that contained at least one keyword from both search groups (PPE and mental health). There were no exclusions for the PPE to any specific types (e.g., surgical face mask, respirator, earmuff, earplug). The exclusion criteria consisted of studies that investigated protective behaviors and psychological health impact as a result of an epidemic or pandemic (e.g., COVID-19, SARS), or those that have investigated the impact of an epidemic or pandemic in the workplace or in general, and editorials, commentaries, reviews, and articles that were not accessible by the primary researcher, or were published in languages other than English. While reviews were not included for assessment, they were examined to identify additional primary studies that may be eligible for inclusion.

### Identification and selection of studies

The primary author searched through the databases and screened the titles for relevant articles to include for review. A second screening was conducted by two other reviewers (K.R.J. and M.M.C.) whereby the titles and abstracts were assessed against the eligibility criteria. The literature was then compared, and duplicate articles were removed. The primary author then screened the full texts of the remaining articles and summarized them into a separate document for discussion with the team. Article relevance was then discussed between the authors (R.L., K.R.J., and M.M.C.) before data was extracted and charted.

### Data extraction and charting

After relevant articles were identified, data was extracted and charted in an Excel spreadsheet. The data that were extracted and recorded included the title of the article, date of publication, name of journal, study population, PPE assessed, data collection tools, risks assessed, and key findings.

### Summarizing the findings

The findings were then summarized into the following research domains: psychological health impact of respiratory PPE and psychological health impact of hearing protection PPE.

## Results

**Fig. 2:**
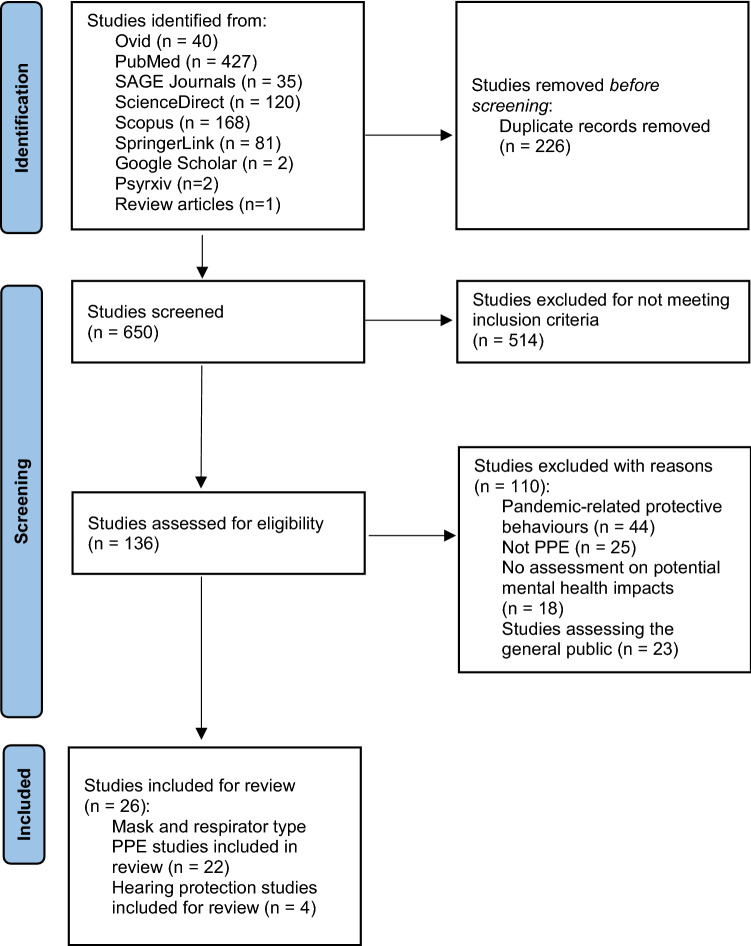
Review schematic of the article search and selection process

A total of 871 articles for mask/respirator-type PPE and hearing protection were retrieved from the selected six databases (Fig. 2 review schematic), plus an additional two (2) articles relevant to hearing protection, which were subsequently retrieved from Google Scholar, two (2) from preprint servers, and one (1) from a review article giving 876 papers. Two hundred and twenty-six (226) duplicate articles were then excluded. Of the remaining 650 articles, 514 articles were excluded for not meeting the inclusion criteria. The remaining 136 articles were then screened by the authors for eligibility for inclusion, of which 110 articles were removed after being deemed not relevant towards the aim of the review, i.e., were primarily focused on pandemic and mask wearing behaviors, the devices being assessed were not PPE (e.g., anesthesia mask, in-ear hearing device), and were articles investigating the general public and not workplaces. The remaining twenty-six (26) articles were deemed relevant and were included for review. From the included articles, twenty-two (22) articles were related to mask and respirator-type PPE, and four articles (4) were relevant for hearing protection. Of the 26 articles, nine (9) were cross-sectional studies, fifteen (15) experimental studies, and two (2) cohort studies (Table 1).

**Table 1:** Characteristics of included studies

Reference; location; study population; sample size	Study design	Risks assessed	PPE assessed
*Mask and respirator articles*			
Bandaru et al. (2020( India; Healthcare (*n* = 20)	Cohort study; speech audiometry	Communication (speech intelligibility)	N95; face shield
Bani et al. (2021) Italy; Healthcare (*n* = 208)	Cross-sectional study; facial emotion recognition task	Communication (emotion recognition)	Surgical mask
Benítez et al. (2020) Multiple countries; Healthcare (*n* = 134)	Cross-sectional study; online survey; study designed questionnaire	Perceived PPE-related impacts; communication; cognitive impact	Surgical mask; N95; face shield
Bottalico et al. (2020) USA; University (n = 40)	Experimental study; speech stimuli (different mask conditions)	Communication (speech intelligibility)	Surgical mask; N95; fabric mask
Cheok et al. (2021) Singapore; Healthcare (*n* = 402)	Cross-sectional study; survey; study designed questionnaire	Perceived PPE-related impacts; communication	Surgical mask; N95; filtering face piece mask; cloth mask/scarf (not PPE)
Choudhury et al. (2020) India; Healthcare (*n* = 75)	Cohort study; physiological variables; perceived exertion questionnaire	Physiological factors (heart rate, oxygen saturation, perfusion index); perceived PPE-related impacts	N95
Emanuel et al. (2017) England; dentistry (*n* = 72)	Cross-sectional study; demographic questionnaire; anxiety questionnaire	Psychological illness (Anxiety)	Surgical mask; transparent visor
Hayirli et al. (2021) USA; Healthcare (*n* = 55)	Cross-sectional study; semi-structured interviews; study designed questions	Communication	Surgical mask; powered air -purifying respirators (PAPR)
Hoedl et al. (2020) Austria; Healthcare (*n* = 2600)	Cross-sectional study; online survey; study designed questionnaire	Psychological factors (stress)	Surgical mask; Filtering face piece mask
İpek et al. (2021) Turkey; Healthcare (n = 34)	Experimental study; study designed questionnaire; blood gas assessment	Perceived PPE-related impacts; physiological factors (blood gas); cognitive impact	N95
Kratzke et al. 2021 USA; Healthcare (*n* = 200)	Experimental study; study designed questionnaire; communication questionnaire	Communication; psychological factors (Empathy, Trust)	Surgical mask; Clear mask
Nguyen et al. (2021) Canada; Healthcare (*n* = 12)	Experimental study; participants performed speech intelligibility tasks with and without in-ear devices; N95, surgical mask, PAPR	Communication (speech intelligibility, listening effort)	Surgical mask; N95; PAPR
Radonovich et al. (2009) USA; Healthcare (n = 16)	Experimental study; modified rhyme test; disposable and reusable respiratory PPE, elastomeric respirator, and powered air-purifying respirator	Communication (speech intelligibility)	Surgical mask; PAPR; N95
Rebmann et al. (2013) USA; Healthcare (*n* = 10)	Experimental study; physiologic variables; perceived exertion questionnaire; perceived thermal comfort questionnaire; perceived N95 comfort questionnaire; subjective symptoms questionnaire; PPE compliance; temperature and relative humidity	Physiological factors (blood pressure, heart rate, CO_2_ and O_2_); Perceived PPE-related impacts	Surgical mask; N95
Sakuma and Ikeda (2021) Japan; University (*n* = 49)	Experimental study; emotional state questionnaire	Psychological factor (interpersonal space)	Surgical mask (and back sunglasses)
Schlögl et al. (2021) Europe and North America; Healthcare (*n* = 226)	Cross-sectional study; online survey; study designed questionnaire	Communication	Face masks in general (did not mention type specifically)
Singh et al. (2021) Multiple countries; healthcare (*n* = 220)	Cross-sectional study; online survey; study designed questionnaire	Communication; perceived PPE-related impacts	Surgical mask; N95; masks with valves
Thiagarajan et al. (2021) India; Healthcare (*n* = 342)	Cross-sectional study; online survey; study designed questionnaire	Psychological factors (fatigue, stress); perceived PPE-related impacts	Surgical mask; N95; face shield mask; face respirators; face shield
Thomas et al. (2011) USA; Emergency medical services (*n* = 4)	Experimental study; speech intelligibility over radio while wearing various masks	Communication (speech intelligibility)	Surgical mask; N95
Tornero-Aguilera and Clemente-Suárez (2021) Spain; University (*n* = 50)	Experimental study; blood oxygen saturation; heart rate and heart rate variability; mental fatigue; reaction time	Cognitive impact, physiological impact (oxygen saturation, heart rate)	Surgical mask
Wong et al. (2013) Hong Kong; Healthcare (*n* = 1030)	Experimental study; patient-rated empathy questionnaire; patient satisfaction; patient enablement	Psychological factors (empathy, satisfaction, enablement)	Surgical mask
Yi et al. (2021) USA; University (*n* = 26)	Experimental study; effects of masks on speech intelligibility in noise	Communication (speech intelligibility)	Surgical mask; transparent mask
*Hearing Protection Articles*			
Dastpaak et al. (2019) Iran; University (*n* = 32)	Experimental study; speech intelligibility while using hearing protection	Communication (speech intelligibility)	Earplug
Karami et al. (2020) Iran; University (*n* = 15)	Experimental study; effect of hearing protection on speech intelligibility	Communication; speech intelligibility	Earplug; Earmuff
Kianmehr et al. (2017) Iran; Stone workers (*n* = 60)	Experimental study; aggression questionnaire; noise intensity; blood pressure; demographic data	Psychological factors (aggression); physiological factors; blood pressure	Earplug; Earmuff
Tavakolizadeh et al. (2019) Iran; Stone workers (*n* = 60)	Experimental study; anxiety questionnaire; depression questionnaire; noise intensity; blood pressure; demographic data	Psychological illness (depression, anxiety); physiological factors (blood pressure)	Earplug; Earmuff

Although there was some variation in the industries and populations represented in the included literature, the healthcare industry was prominent, with fewer from other industries (Table 2). The studies were from a wide range of countries, with the majority from the United States of America (USA) (7 articles, 27%), followed by Iran (4 articles, 15%). Further details of article origin can be found in Table 1 and relevant findings can be found in Supplementary Table 2.

**Table 2:** Industries studied (*n* = 26)

Industry/participant group	Count	Percentage (%)
Healthcare	16	61
University	6	23
Stone workers	2	8
Dentistry	1	4
Emergency medical service	1	4

### Impact of PPE

Most studies evaluated more than one psychosocial hazard or stressor associated with the use of PPE. Of the stressors assessed, over half of the articles assessed the impact of PPE on communication (16 articles, 62%) (Bandaru et al. 2020; Bani et al. 2021; Benítez et al. 2020; Bottalico et al. 2020; Brown et al. 2021; Cheok et al. 2021; Dastpaak et al. 2019; Hayirli et al. 2021; Karami et al. 2020; Kratzke et al. 2021; Nguyen et al. 2021; Radonovich Jr et al. 2009; Schlögl et al. 2021; Singh et al. 2021; Thomas et al. 2011; Yi et al. 2021). Most of these articles assessed the impact on speech intelligibility, facial and emotion recognition, and listening effort when PPE is worn. Studies also evaluated the impact on psychological health factors, such as stress, fatigue, and emotional state (5 articles, 19%) (Hoedl et al. 2020; Kratzke et al. 2021; Sakuma and Ikeda 2021; Thiagarajan et al. 2021; Wong et al. 2013). Two articles investigated the effect of PPE use on psychological illness symptoms, specifically anxiety and depression (2 articles, 8%) (Emanuel et al. 2017; Tavakolizadeh et al. 2019). A small number of articles addressed the potential cognitive impact on those who use PPE, including concentration, reaction time and decision-making (3 articles, 6%) (Benítez et al. 2020; İpek et al. 2021; Tornero-Aguilera and Clemente-Suárez, 2021). The remaining studies assessed psychosocial stressors generally, including physical symptoms and other perceived subjective impacts related to PPE which we have grouped together as ‘perceived PPE-related impacts’ (7 articles, 27%) (Benítez et al. 2020; Cheok et al. 2021; Choudhury et al. 2020; İpek et al. 2021; Rebmann et al. 2013; Singh et al. 2021; Thiagarajan et al. 2021).

### Psychological impact of masks and respirators

There were fifteen articles related to communication impacts associated with respiratory PPE use (Bandaru et al. 2020; Bani et al. 2021; Benítez et al. 2020; Bottalico et al. 2020; Cheok et al. 2021; Hayirli et al. 2021; Kratzke et al. 2021; Nguyen et al. 2021; Radonovich Jr et al. 2009; Ruba and Pollak, 2020; Schlögl et al. 2021; Singh et al. 2021; Thibodeau et al. 2021; Thomas et al. 2011; Yi et al. 2021). These studies specifically investigated communication in general, emotion and facial recognition, listening effort, sentence recall, and speech intelligibility. Most articles reported that there was a negative impact on communication between individuals when facial PPE was worn (10 articles, 67% of 15 articles). Like the previously discussed hearing protection related studies, impacts on speech intelligibility become more apparent in high levels of background noise, while in low background noise, masks had little to no effect on speech intelligibility (Thomas et al. 2011). Furthermore, research that tested the effect of transparent masks reported comparable findings (Kratzke et al. 2021). Transparent masks were reported to alleviate communication impacts, especially in those with hearing impairments.

There were seven articles that reported individuals’ perceived PPE impacts (Benítez et al. 2020; Cheok et al. 2021; Choudhury et al. 2020; İpek et al. 2021; Rebmann et al. 2013; Singh et al. 2021; Thiagarajan et al. 2021). These articles reported on the physical and subjective symptoms associated with wearing PPE. Additionally, there were three articles that assessed the cognitive impact associated with facial PPE with two studies finding that participants experienced a negative effect on their decision-making and felt greater attention deficit and difficulty concentrating (Benítez et al. 2020; İpek et al. 2021). However, research by Tornero-Aguilera and Clemente-Suárez (2021) did not report an impact on cognition, specifically mental fatigue and reaction time. Though this may be due to the different cohorts being studied with the previous two articles evaluating those in healthcare compared to the latter investigating university students.

Of the twenty-two articles related to mask and/or respirator-type PPE, only one study assessed the effect of PPE use on psychological illness symptoms of those who interacted with those wearing PPE (Emanuel et al. 2017). This study reported that those who interacted with those who wore PPE experienced negative impacts on their psychological health, with increasing levels of anxiety. Additionally, four articles assessed for psychological factors and described similar findings (Kratzke et al. 2021; Sakuma and Ikeda 2021; Thiagarajan et al. 2021; Wong et al. 2013). PPE that occluded a part of the face resulted in negative psychological impacts, specifically increasing stress, fatigue, and perceiving someone who wore PPE in a more negative light. Of note, Hoedl et al. (2020) reported no significant association between stress and PPE use in nurses, but nurses who wore masks for more than eight hours had significantly higher levels of stress than those who used masks for shorter periods (*p* = 0.000).

### Psychological impact of hearing protection

Two of the four hearing protection studies investigated impacts on communication, specifically on how hearing protection effected speech intelligibility (Dastpaak et al. 2019; Karami et al. 2020). Both articles reported that the higher the background noise the more it affected speech intelligibility regardless of whether hearing protection devices were worn or not. Furthermore, both papers reported that the use of some hearing protectors was effective in improving speech intelligibility in the presence of background noise.

Two of the articles discussed potential psychological impacts on stone workers who wore hearing protection. One study (Tavakolizadeh et al. 2019) used an intervention to investigate the effects of hearing protection use on anxiety and depression. The study found that anxiety significantly decreased after using hearing protection devices (HPD), with earplugs reducing symptoms of anxiety more than earmuffs. Earplug use was also associated with a significant decrease in depression (*p* < 0.01). But earmuff use was associated with a significant increase in depression (*p* < 0.01). The other article evaluated the effect of earmuffs and earplugs use on aggression in stone workers (Kianmehr et al. 2017). The findings of this study revealed that earplugs have a protective effect, having a significantly higher contribution to reducing aggression, specifically physical aggression, verbal aggression, anger, and hostility, than earmuffs (*p* < 0.05).

Six (6) articles also assessed physiological impacts. However, as this review is primarily focused on the psychological impact of PPE use, the physiological impact will not be further discussed as it is not within the scope of this review.

## Discussion

To our knowledge, this is the first scoping review to focus on and collate PPE-related impacts on the psychological health of those who either wear it or are interacting with someone who has donned PPE, specifically mask and respirator-type PPE and hearing protectors. The findings of this review suggest that the use of masks, respirators or hearing protection can act as a psychosocial hazard leading to a stress response and negative psychological health consequences.

### Mask and respirator use

#### Impact on psychological health

Findings for mask and respirator-type PPE suggest that there is a potential negative impact on psychological health and therefore the use of this PPE is a psychosocial hazard. Studies reviewed, alongside other similar studies that were excluded from assessment, reported that there were negative impacts on psychological health for both the wearer and for those who interacted with the wearer with increased levels of anxiety, depression, and psychological distress (Biermann et al. 2021; Emanuel et al. 2017; Homans and Vroegop 2021; Saunders et al. 2021; Wu et al. 2011). The study by Emanuel et al. (2017) assessing the effect of dental clinicians wearing masks and visors on special care patients, found that the style of face protection had an effect on the patients’ level of anxiety. These findings suggest that the more occluded a face becomes, the more anxious other people in close proximity become. Findings from several studies investigating the effect of masked and unmasked conditions or different types of masks, including transparent masks, have reported comparable results (Kratzke et al. 2021; Marini et al. 2021; Wong et al. 2013). These studies all demonstrated that facial occlusion of the lower face resulted in more negative psychological implications, in terms of feelings of trust and empathy towards the person wearing PPE, when compared to unmasked conditions or when transparent masks were used.

Though it should be mentioned that studies have observed cultural differences regarding protective behaviors, such as the use of PPE. A study by Wang et al. (2020) found cultural differences between Chinese and Polish citizens between physical, mental health, and mask use during COVID-19. Their study reported that Chinese were more accepting of face masks compared to Poles due to cultural differences. The same applied to respirators used to protect those who work in the industries that require them, such as mining. These cultural differences may also carry over to the workplace which may influence workers’ acceptance of PPE and subsequently their ability to cope with PPE-related stressors.

#### Impact on subjective and physical symptoms

Subjective and physical symptoms associated with PPE use, such as discomfort could possibly be a psychosocial stressor. A large proportion of the studies investigated reported subjective and physical impacts to individuals, including headaches, breathing difficulties, fatigue, and discomfort (Benítez et al. 2020; Cheok et al. 2021; Choudhury et al. 2020; İpek et al. 2021; Karagkouni, 2021; Rebmann et al. 2013; Ribeiro et al. 2020; Singh et al. 2021; Thiagarajan et al. 2021). Findings from a study by Thiagarajan et al. (2021) investigating PPE use and comfort levels among surgeons during the COVID-19 pandemic, found that N95 masks and eye protection contributed most to surgeon discomfort, specifically headache, dryness of mouth, breathing difficulty and fogging of eye protection, which were related to reported increased stress and fatigue. This raises concerns because symptoms such as headaches could have a negative effect on concentration and work performance (Hajjij et al. 2020).

#### Impact on cognition

Of the studies reviewed, three articles assessed cognitive impacts on those who wore masks or respirators (Benítez et al. 2020; İpek et al. 2021; Tornero-Aguilera and Clemente-Suárez 2021). Two articles reported that there was an impact to those in the healthcare profession (i.e., surgeons, nurses), specifically respondents reported they felt that PPE had an influence on their decision-making process (Benítez et al. 2020) and felt significantly greater attention deficit and difficulty concentrating (İpek et al. 2021). It is possible that subjective and physical responses (e.g., headache, discomfort, etc.) associated with mask or respirator use could potentially lead to an increase in occupational stress, which in turn has an impact on one’s cognition. Stress has been documented to have a negative effect on working memory, attention, response inhibition and cognitive flexibility (Girotti et al. 2018). In the workplace, this translates to an impairment in an individual’s ability to concentrate, remember, plan, and control their impulses. Consequently, this could lead to further stressful situations that, if not managed, could increase one’s risk of psychological illness or other injuries.

### Hearing protection use

#### Impact on psychological health

While there seems to be evidence of potential negative impacts on an individual’s psychological health when using masks or respirators, the same could not be said for hearing protection. Our retrieval of hearing protection studies related to psychological health only yielded two articles (Kianmehr et al. 2017; Tavakolizadeh et al. 2019). Both articles assessed the impact on psychological symptoms such as anxiety and depression. Psychological symptoms, such as anxiety and depression, are often characterized by impaired social behaviors including excessive aggression, anger, and violence (Barrett et al. 2013; Chung et al. 2019; Meyrueix et al. 2015; Neumann et al. 2010). Findings revealed that the use of earplugs had a protective effect on these symptoms in stone workers (Kianmehr et al. 2017; Tavakolizadeh et al. 2019). Earmuffs were also found to reduce anxiety and aggression in stone workers, though were not as effective when compared to earplugs. However, depression was found to increase in those who wore earmuffs (Tavakolizadeh et al. 2019). Possible explanations for the difference include that earmuffs are large, cumbersome, unsuitable in warmer environments and in this study had higher attenuation than earplugs, which could block out more background noise (environmental noises and voices).

### Communication impacts from mask, respirator or hearing protection use

An impairment in communication can lead to miscommunication between peers and supervisors that could have an influence on stress and negative emotions. Such issues, if left unresolved, could lead to conflicts or worsen a situation. Studies have found that within work environments unsolved conflicts can be detrimental to all parties involved by increasing risk of stress, fatigue and emotional exhaustion (Bültmann et al. 2002; Danielsson et al. 2015; Grandey et al. 2007). Facial occlusion may also have an impact on verbal and nonverbal communication. The majority of evidence from the studies reviewed reported negative impacts on communication, specifically emotion and facial recognition (Bani et al. 2021; Ruba and Pollak 2020), speech intelligibility (Bottalico et al. 2020; Nguyen et al. 2021; Radonovich Jr et al. 2009; Thomas et al. 2011), speech perception and listening effort (Bandaru et al. 2020), and communication in general (Benítez et al. 2020; Cheok et al. 2021; Hayirli et al. 2021; Kratzke et al. 2021; Schlögl et al. 2021; Singh et al. 2021). In situations where the use of masks or respirators are needed there may be an increased risk of stress due to partial facial occlusion and impaired communication with others. Of the few studies that investigated speech intelligibility, a study by Thomas et al. (2011) found that in low to no noise environments, masks had little to no effect on speech intelligibility. However, like hearing protection studies, background noise was found to have a significant impact on speech intelligibility regardless of the presence of PPE. Though that is not to say that PPE has no or less impact on communication, but background noise may have a synergistic effect with PPE attenuation that impairs communication, depending on the equipment used. For example, the results from a study by Toscano and Toscano (2021) revealed that in high background noise differences between masks become more apparent. Similarly, findings from Thomas et al. (2011) revealed that from within a loud environment (helicopter cockpit while the engine is operational) only some of the masks tested maintained higher accuracy of recited words.

In our scoping review we included two articles assessing impacts on communication when wearing hearing protectors, which specifically measured speech intelligibility (Dastpaak et al. 2019; Karami et al. 2020). Similar findings were reported between both articles which demonstrated that the higher the background noise the more it impacted speech intelligibility regardless of whether hearing protection was worn or not. Furthermore, the articles reported that the use of hearing protection, while in the presence of background noise, was effective in improving speech intelligibility (Dastpaak et al. 2019; Karami et al. 2020). In comparison, findings from Rocha et al. (2021) did not observe such effects with hearing protection. While they too found evidence of speech intelligibility decreasing when wearing HPDs in high levels of noise, their study did not report an improvement in speech intelligibility. Moreover, in their study, the greatest negative impact of hearing protectors had affected individuals with normal hearing, who were not normally exposed to occupational noise, suggesting that hearing protectors do not always improve speech intelligibility. In addition, evidence suggested that an increase in the attenuation of the hearing protectors led to an increase in speech interference (Karami et al. 2020). Together, these findings demonstrate the importance of matching the hearing protection to the work environment to maximize speech intelligibility. While the use of hearing protection with a high noise reduction rating (NRR) may protect the user from noise exposure, this overprotection can result in workers being exposed to other workplace stressors like impaired communication.

This review has identified possible evidence of PPE use acting as a psychosocial hazard or stressor that could have a negative impact on an individual’s psychological health. The most studied industry in relation to this topic was healthcare. Understandably, this is an important industry to evaluate as patients’ lives are in the hands of healthcare professionals and the quality of the healthcare workers’ psychological health is critical. However, in light of the current COVID-19 pandemic, the use of PPE has increased and become stricter in certain workplaces. This sudden change to everyone’s daily lifestyle and workplace behaviors has shed light on the potential stressors associated with the use of PPE that has yet to be fully explored. Hence, future research should investigate the use of PPE and its impact on occupational stress and psychological health in other industries outside of healthcare. In addition, studies that were included for review were either cross-sectional or experimental and could not evaluate long-term effects on psychological health. Future research should consider longitudinal study designs to investigate effects of long-term use of PPE.

### Limitations

This scoping review employed a systematic search strategy of the scientific literature to identify studies on psychological health impacts due to PPE usage. Preliminary research into the topic revealed that this was a topic that has yet to be fully explored. Therefore, the selection criteria used were quite broad with the aim of retrieving as many relevant studies as possible. However, we acknowledge that the articles retrieved may underrepresent the research. Our review only included studies in English and may have missed findings reported in other languages. The included studies were conducted in multiple countries, in various industries and populations, and at different times before and during COVID-19, which may have influenced reported findings. A difference in cultures has been found to influence perceptions and behaviors surrounding PPE and protective behaviors (Wang et al. 2020). Finally, hearing protection related articles included for review were limited in number and the conclusions drawn may not be entirely accurate.

## Conclusion

PPE is commonly used in workplaces to protect workers from physical, chemical, and biological hazards. However, the use of PPE could pose hidden risks for users, with this review identifying possible negative impacts on worker’s psychological health. The findings suggest that the use of masks and respirators may lead to an increase in occupational stress and risk of psychological symptom development. In comparison, in two studies hearing protection was found to have protective effects in reducing psychological symptoms and increasing speech intelligibility. These findings demonstrate the importance of considering the psychosocial hazards that may be created by using masks or respirators in the workplace as part of designing occupational health hazard control strategies.

## Supplementary Information

Below is the link to the electronic supplementary material.

Supplementary file1 (XLSX 10 kb)

Supplementary file2 (DOCX 41 kb)
