# Supplementary material for: The contribution of respiratory and hearing protection use to psychological distress in the workplace: a scoping review
Source: Int Arch Occup Environ Health. 2022 Apr 26;95(8):1647–59. doi: 10.1007/s00420-022-01863-7 (PMC9041289; doi:10.1007/s00420-022-01863-7)
Supplement: Supplementary file 2 — Supplementary file2 (DOCX 41 kb) [file 420_2022_1863_MOESM2_ESM.docx]

| **Reference** | **Location** | **Sample size** | **Industry** | **Data collection method** | **Risks assessed** | **Findings on PPE with respect to mental health** |
| --- | --- | --- | --- | --- | --- | --- |
| **Mask and respirator articles** | | | | | | |
| Bandaru, S. V., Augustine, A. M., Lepcha, A., Sebastian, S., Gowri, M., Philip, A., & Mammen, M. D. (2020). The effects of N95 mask and face shield on speech perception among healthcare workers in the coronavirus disease 2019 pandemic scenario. J Laryngol Otol, 134(10), 895-898. <doi:https://doi.org/10.1017/S0022215120002108> | India | 20 | Healthcare | - Cohort study   - Speech perception with N95 masks and face shields - Speech audiometry | - Communication   - Speech intelligibility | - There was a significant increase in speech reception threshold and decrease in speech discrimination score when using N95 mask and face shield |
| Bani, M., Russo, S., Ardenghi, S., Rampoldi, G., Wickline, V., Nowicki, S., & Strepparava, M. G. (2021). Behind the Mask: Emotion Recognition in Healthcare Students. Medical Sci. Educ., 1-5. <doi:https://doi.org/10.1007/s40670-021-01317-8> | Italy | 208 | Healthcare | - Cross-sectional study - Facial emotion recognition task   - Diagnostic Analysis of Nonverbal Accuracy 2 – Adult Faces (DANVA2-AF)   - Masked vs Unmasked | - Communication   - Emotion recognition | - Emotions are more difficult to recognise in masked faces than unmasked faces - There were more misattributions for happy, sad and angry masked faces, but not fearful faces |
| Benítez, C. Y., Güemes, A., Aranda, J., Ribeiro, M., Ottolino, P., Di Saverio, S., . . . Ramos, J. P. (2020). Impact of personal protective equipment on surgical performance during the COVID-19 pandemic. *World J. Surg., 44*(9), 2842-2847. <doi:https://doi.org/10.1007/s00268-020-05648-2> | Multiple countries | 134 | Healthcare | - Cross-sectional study - Online survey - Study designed questionnaire   - Demographic questions   - PPE impact questions   - Surgical fatigue | - Perceived PPE related impacts - Communication - Cognitive impact | - More than half the respondents felt their surgical performance was hampered by COVID-19 PPE - Less than half felt protected with the use of PPE, and the same proportion of people felt that the PPE influenced their decision making - Majority of respondents complained of reduced comfort and experienced increased surgical fatigue - More than half of the respondents complained of visual impairment and communication difficulties |
| Bottalico, P., Murgia, S., Puglisi, G. E., Astolfi, A., & Kirk, K. I. (2020). Effect of masks on speech intelligibility in auralized classrooms. *J. Acoust. Soc. Am., 148*(5), 2878-2884. <doi:https://doi.org/10.1121/10.0002450> | USA | 40 | University | - Experimental study   - Participants listened to stimuli online of different face masks - Speech stimuli   - Four mask conditions per two-room acoustic conditions | - Communication   - Speech intelligibility | - Fabric masks had the highest levels of attenuation, while N95 and surgical masks had lower levels - Each of the masks were found to have a negative impact on speech intelligibility |
| Cheok, G. J. W., Gatot, C., Sim, C. H. S., Ng, Y. H., Tay, K. X. K., Howe, T. S., & Koh, J. S. B. (2021). Appropriate attitude promotes mask wearing in spite of a significant experience of varying discomfort. *Infection, Disease & Health, 26*(2), 145-151. <doi:https://doi.org/10.1016/j.idh.2021.01.002> | Singapore | 402 | Healthcare | - Cross-sectional study - Survey - Study designed questionnaire   - Demographic questions   - PPE related questions   - Perceived necessity   - Level of discomfort | - Perceived PPE related impacts - Communication | - Nearly 70% of the respondents thought that wearing masks was essential in controlling the spread of the virus - Most common mask associated discomforts were difficulty breathing, communication and fogging of spectacles, with certain demographic groups - Respondents who wore masks for longer hours each day reported higher levels of discomfort, dermatological issues and sweating - Younger respondents reported higher incidence of dermatological issues and sweating |
| Choudhury, A., Singh, M., Khurana, D. K., Mustafi, S. M., Ganapathy, U., Kumar, A., & Sharma, S. (2020). Physiological effects of N95 FFP and PPE in healthcare workers in COVID intensive care unit: A prospective cohort study. *Indian J Crit Care Med, 24*(12), 1169. <doi:https://doi.org/10.5005/jp-journals-10071-23671> | India | 75 | Healthcare | - Cohort study   - Physiological effects of N95 masks - Physiological variables   - Heart rate   - Oxygen saturation   - Perfusion index - Perceived exertion questionnaire   - Borg CR10 scale (Foster et al., 20010 | - Physiological factors   - Heart rate   - Oxygen saturation   - Perfusion index - Perceived PPE related impacts | - Rating of perceived exertion score indicated increased discomfort with continuous use of N95 mask - Major adverse effects reported were fogging, headache, tiredness, difficulty in breathing, and mask soakage with sweat - Significant increase in heart rate with prolonged N95 use |
| Emanuel, R., Corcoran, R., & Cass, H. (2017). A study of special care dental patient preference toward choice of mask and visor use by dental clinicians. *Spec Care Dentist, 37*(4), 164-167. <doi:https://doi.org/10.1111/scd.12226> | England | 72 | Dentistry | - Cross-sectional study   - Questionnaire questions related to video clips - Demographic questionnaire - Anxiety questionnaire   - Modified Dental Anxiety Scale (MDAS) | - Mental health   - Anxiety | - Majority of patients (68%) prefer the “visor only” choice, followed by “mask only” and “visor and mask” combination - Style of face protection would affect the levels of anxiety |
| Hayirli, T. C., Stark, N., Bhanja, A., Hardy, J., Peabody, C. R., & Kerrissey, M. J. (2021). Masked and distanced: a qualitative study of how personal protective equipment and distancing affect teamwork in emergency care. *Int J Qual Health Care, 33*(2), mzab069. <doi:https://doi.org/10.1093/intqhc/mzab069> | USA | 55 | Healthcare | - Cross-sectional study - Semi-structured interviews - Study designed questions   - Demographic questions   - Experiences with COVID-19 in terms of teamwork and communication   - How pandemic affected their work | - Communication | - Material barriers related to wearing masks, gowns and powered air-purifying respirators was found to be a challenge to teamwork - Material barriers disrupted communication, roles, and interpersonal relationships - Material barriers muffled information flow, impeded team member recognition and tole/task division, and reduced belonging and cohesion while increasing interpersonal strain |
| Hoedl, M., Eglseer, D., & Bauer, S. (2020). Associations between personal protective equipment and nursing staff stress during the COVID-19 pandemic. *medRxiv*. | Austria | 2600 | Healthcare | - Cross-sectional study - Online survey - Study designed questionnaire based on Donabedian’s quality of health care model (1966)   - PPE related questions   - Perceived Stress Scale (PSS)   - Demographic questions | - Psychological factors   - Stress | - More than two-thirds of the participants had moderate or even high-perceived stress levels - There was no significant association between the use of PPE and stress - Nurses who wore masks for more than 8hrs had significantly higher stress levels than those who used masks for shorter periods |
| İpek, S., Yurttutan, S., Güllü, U. U., Dalkıran, T., Acıpayam, C., & Doğaner, A. (2021). Is N95 face mask linked to dizziness and headache? *Int Arch Occup Environ Health*, 1-10. <doi:https://doi.org/10.1007/s00420-021-01665-3> | Turkey | 34 | Healthcare | - Experimental study   - Participants wore mask for 1-4hrs (surgical mask and N95 mask) - Study designed questionnaire   - Demographic questions   - Physical symptoms   - Psychosomatic symptoms - Blood gas assessment   - Radiometer ABL800 FLEX | - Perceived PPE related impacts - Physiological factors   - Blood gas - Cognitive impact | - Results from capillary blood gas pH indicated alkalosis and CO2 was significantly low in those using N95 masks - The use of N95 masks were found to significantly increase the rates of headaches, respiratory distress, drowsiness, and feeling of numbness compared to surgical masks - Respondents reported that they felt significantly greater attention deficit and difficulty concentrating with N95 masks compared to surgical masks |
| Kratzke, I. M., Rosenbaum, M. E., Cox, C., Ollila, D. W., & Kapadia, M. R. (2021). Effect of Clear vs Standard Covered Masks on Communication With Patients During Surgical Clinic Encounters: A Randomized Clinical Trial. *JAMA Surg, 156*(4), 372-378. <doi:https://doi.org/10.1001/jamasurg.2021.0836> | USA | 200 | Healthcare | - Experimental study   - Clear vs covered mask - Study designed questionnaire   - Demographic data obtained from health record   - Empathy and trust   - Rating on mask worn - Communication questionnaire   - Clinician and Group Consumer Assessment of Healthcare Providers and Systems (CG-CAHPS) | - Communication - Psychological factors   - Empathy   - Trust | - Majority of respondents rated surgeons higher for providing understandable explanations, demonstrating empathy, and building trust when the clear mask was worn - Respondents preferred clear masks because of improved surgeon communication and appreciation for visualisation of the face |
| Mendel, L. L., Gardino, J. A., & Atcherson, S. R. (2008). Speech understanding using surgical masks: a problem in health care? *J Am Acad Audiol, 19*(9), 686-695. <doi:https://doi.org/10.3766/jaaa.19.9.4> | USA | 31 | General public (impaired hearing) | - Experimental study   - Participants listened to recordings and repeated what they heard - Conditions: surgical mask and no mask | - Communication   - Speech intelligibility | - Spectral analyses revealed significant difference between masked and unmasked - There were no detrimental effects on speech intelligibility for the participants in the masked condition |
| Nguyen, D. L., Kay-Rivest, E., Tewfik, M. A., Hier, M., & Lehmann, A. (2021). Association of In-Ear Device Use With Communication Quality Among Individuals Wearing Personal Protective Equipment in a Simulated Operating Room. *JAMA NETW OPEN, 4*(4), e216857-e216857. <doi:https://doi.org/10.1001/jamanetworkopen.2021.6857> | Canada | 12 | Healthcare | - Experimental study   - Participants performed speech intelligibility tasks with and without in-ear communication devices   - Simulated operating theatre environment   - Participants did suturing task during speech recognition task   - Talker wore 3 types of PPE (N95, surgical mask, powered air-purifying respirator PAPR)   - Communication device (SonX) - Speech intelligibility   - Modified Rhyme Test (MRT)   - AzBio Sentence Test - Listening effort   - NASA Task Loader Index (NASA-TLX) | - Communication   - Speech intelligibility | - While wearing an N95 mask, mean speech intelligibility was 98.8% without an in-ear device and 94.3% with the device - While wearing the surgical face mask, the mean speech intelligibility was 58.5% without the in-ear device vs 90.8% with the device. - Use of devce associated wut decreased listening effort - While wearing PAPR, the mean speech intelligibility was 84.6% without the in-ear device vs 94.5% with the device - A significant improvement in speech intelligibility was associated with the surgical face mask and PAPR |
| Radonovich Jr, L. J., Yanke, R., Cheng, J., & Bender, B. (2009). Diminished speech intelligibility associated with certain types of respirators worn by healthcare workers. *J Occup Environ Hyg, 7*(1), 63-70. <doi:https://doi.org/10.1080/15459620903404803> | USA | 16 | Healthcare | - Experimental study   - Modified rhyme test - Conditions (disposable and reusable respiratory PPE, elastomeric respirator, and powered air-purifying respirator) | - Communication   - Speech intelligibility | - While not all results were statistically significant, respirators decreased speech intelligibility by a range of 1%-17% - Elastomeric respirators with voice augmentation equipment were associated with higher speech intelligibility than models without it - Hearing clarity while wearing powered air-purifying respirator was lower than without |
| Rebmann, T., Carrico, R., & Wang, J. (2013). Physiologic and other effects and compliance with long-term respirator use among medical intensive care unit nurses. *Am. J. Infect. Control, 41*(12), 1218-1223. <doi:https://doi.org/10.1016/j.ajic.2013.02.017> | USA | 10 | Healthcare | - Experimental study   - Physiological and subjective effects of long-term respiratory protection - Physiologic variables   - Blood pressure   - Heart rate   - CO2 and O2 (SenTec CO2 and O2 saturation sensor) - Perceived exertion questionnaire   - Borg Rating of Perceived Exertion Scale - Perceived thermal comfort questionnaire   - Frank Scale of Perceived Thermal Comfort Scale - Perceived N95 comfort questionnaire   - Roberge Respirator Comfort Scale - Subjective symptoms questionnaire   - Roberge Subjective Symptoms During Work Scale - PPE compliance - Temperature and relative humidity | - Physiological factors   - Blood pressure   - Heart rate   - CO2 and O2 - Perceived PPE related impacts | - Compared to baseline transcutaneous CO2 levels significantly increased, especially when comparing N95 mask to surgical mask - Perceived exertion, perceived shortness of air, headaches, light-headedness, and difficulty communicating also increased over time - Almost one-quarter of mask removals were due to discomfort |
| Sakuma, N., & Ikeda, K. (2021). Effects of Modulated Emotion and Facial Masking Encounter on Personal Distance. *Percept Mot Skills, 128*(1), 178-190. <doi:https://doi.org/10.1177/0031512520973515> | Japan | 49 | University | - Experimental study   - Stop-distance task (uncomfortable feeling dependent on distance from one another)   - Positive vs negative emotion conditions   - Masked (face mask and sunglasses) vs unmasked - Emotional state questionnaire   - Ohtomo et al., 2010 | - Psychological factor   - Interpersonal space | - The participants personal space increased for the “masked” condition more than the “unmasked” condition, which was common for both the positive and negative emotion conditions |
| Schlögl, M., Singler, K., Martinez-Velilla, N., Jan, S., Bischoff-Ferrari, H. A., Roller-Wirnsberger, R. E., . . . Gordon, A. L. (2021). Communication during the COVID-19 pandemic: evaluation study on self-perceived competences and views of health care professionals. Eur Geriatr Med, 1-10. <doi:https://doi.org/10.1007/s41999-021-00532-1> | Europe and North America | 226 | Healthcare | - Cross-sectional study - Online survey - Study designed questionnaire   - Demographic questions   - PPE related impacts and issues   - COVID-19 communication related impacts and issues | - Communication | - Respondents reported that acknowledging emotions and providing information using clear, specific, unambiguous, and consistent lay language while wearing a mask were among the main communications challenges during COVID-19 |
| Singh, P., Bhandoria, G., & Maheshwari, A. (2021). Pharmacological Prophylaxis and Personal Protective Equipment (PPE) Practices in Gynecological Cancer Surgery During COVID-19 Pandemic. *Indian journal of gynecologic oncology, 19*(1), 1-7. <doi:https://doi.org/10.1007/s40944-021-00500-4> | Multiple countries | 220 | Healthcare | - Cross-sectional study - Online survey - Study designed questionnaire   - Demographic questions   - PPE questions   - COVID-19 screening protocol questions | - Communication - Perceived PPE related impacts | - Majority of respondents have reported some degree of discomfort during surgery due to PPE, with some reporting moderate to severe - Most common difficulties were problems in communication and breathing |
| Thiagarajan, S., Shetty, P., Gulia, A., Prakash, G., Pramesh, C. S., & Puri, A. (2021). A Survey of Personnel Protective equipment’s (PPE) Use and Comfort Levels Among Surgeons During Routine Cancer Surgery in the COVID-19 Pandemic. *Indian Journal of Surgical Oncology*, 1-9. <doi:https://doi.org/10.1007/s13193-021-01316-6> | India | 342 | Healthcare | - Cross-sectional study - Online survey - Study designed questionnaire   - Demographic questions   - PPE related questions   - Discomfort and fatigue related to PPE questions | - Psychological factors   - Fatigue   - Stress - Perceived PPE related impacts | - N95 masks and eye protection contributed the most to surgeon discomfort - Headache, dryness of mouth, breathing difficulty/suffocation, and fogging (of eye protection) were the most common reasons for increased stress and fatigue |
| Thomas, F., Allen, C., Butts, W., Rhoades, C., Brandon, C., & Handrahan, D. L. (2011). Does wearing a surgical facemask or N95-respirator impair radio communication? *Air Med. J., 30*(2), 97-102. <doi:https://doi.org/10.1016/j.amj.2010.12.007> | USA | 4 | Emergency medical serices | - Experimental study   - Speech intelligibility over radio while wearing surgical mask and 6 different N95 masks with and without the engine operating | - Communication   - Speech intelligibility | - When the engine was off, there was 100% accuracy regardless of whether a mask was worn or not - When the engine was on, the surgical mask and only two N95 masks maintained 100% accuracy |
| Tornero-Aguilera, J. F., & Clemente-Suárez, V. J. (2021). Cognitive and psychophysiological impact of surgical mask use during university lessons. *Physiol. Behav., 234*, 113342. <doi:https://doi.org/10.1016/j.physbeh.2021.113342> | Spain | 50 | University | - Experimental study   - Masks vs no mask during lectures - Blood oxygen saturation   - Oximeter OXYM4000 - Heart rate and heart rate variability   - Polar V800 - Mental fatigue   - Redondo-Florenz et al.,2020) - Reaction time | - Cognitive impact - Physiological impact   - Oxygen saturation   - Heart rate | - Masks produced an increased heart rate and decreased blood oxygen saturation, but did not significantly decrease more than non-masked condition - Use of surgical mask did not significantly affect mental fatigue perception, reaction time, frequency and nonlinear heart rate variability domains |
| Wong, C. K. M., Yip, B. H. K., Mercer, S., Griffiths, S., Kung, K., Wong, M. C.-s., . . . Wong, S. Y.-s. (2013). Effect of facemasks on empathy and relational continuity: a randomised controlled trial in primary care. *BMC Fam. Pract., 14*(1), 1-7. <doi:https://doi.org/10.1186/1471-2296-14-200> | Hong Kong | 1030 | Healthcare | - Experimental study   - Face mask vs no mask - Patient-rated empathy questionnaire   - Consultation and Relational Empathy (CARE) - Patient satisfaction - Patient enablement   - Patient Enablement Instrument (PEI) | - Psychological factors   - Empathy   - Satisfaction   - Enablement | - A significantly negative effect was found in the patients’ perception of the doctors’ empathy for the masked group - The effect of doctors’ mask wearing was more pronounced in more established doctor-patient relationships - Could have an effect on relational continuity |
| Yi, H., Pingsterhaus, A., & Song, W. (2021). Effects of Wearing Face Masks While Using Different Speaking Styles in Noise on Speech Intelligibility During the COVID-19 Pandemic. *Frontiers in Psychology, 12*, 682677-682677. Retrieved from <https://pubmed.ncbi.nlm.nih.gov/34295288> | USA | 26 | University students | - Experimental study   - Effects of wearing masks on speech intelligibility in presence of background noise - Stimuli   - Target sentences were video recorded   - No face mask, surgical mask, transparent mask   - Clear vs conversation speech   - Background noise conditions: Speech-Shaped Noise (SSN), four-talker babble | - Communication   - Speech intelligibility | - Adverse effects found on surgical masks on speech intelligibility in presence of background noise - Use of face masks decreases speech intelligibility compared to mask in presence of background noise - White noise was found to be less challenging compared to informational maskers (talker babble) - Transparent masks were found to facilitate the ability to comprehend the target sentences by providing individuals with visual information - Clear speech was found to alleviate challenging communication situations, and could compensate impaired visual cues and acoustic signals |
| **Hearing protection articles** | | | | | | |
| Dastpaak, H., Alimohammadi, I., jalal Sameni, S., Abolghasemi, J., & Vosoughi, S. (2019). Effects of earplug hearing protectors on the intelligibility of Persian words in noisy environments. *Appl Acoust, 148*, 19-22. <doi:https://doi.org/10.1016/j.apacoust.2018.11.017> | Iran | 32 | University students | - Experimental study   - Speech intelligibility while using earplug hearing protectors   - 25 Noise Reduction Rating (NRR) earplugs and 32 NRR - Hearing conditions   - Without noise or earplugs   - With noise but without earplugs   - With noise and 25 NRR earplugs - With noise and 32 NRR earplugs | - Communication   - Speech intelligibility | - Hearing protectors improve average value of intelligibility - In the presence of background noise, hearing protectors increase speech intelligibility - The lower the NRR, the higher the intelligibility |
| Karami, M., Aliabadi, M., Golmohammadi, R., & Nahrani, M. H. (2020). The effect of hearing protection devices on speech intelligibility of Persian employees. *BMC Res. Notes, 13*(1), 1-6. <doi:https://doi.org/10.1186/s13104-020-05374-x> | Iran | 15 | University students | - Experimental study   - Effect of hearing protection on speech intelligibility - Three different earmuffs, three different earplugs, and one prototype moulded earplug | - Communication   - Speech intelligibility | - Hearing protection at S/N = 0 showed higher effect on improving speech intelligibility compared to S/N = +5 - Increasing noise attenuation led to an increase in speech interference |
| Kianmehr, M., Tavakolizadeh, J., Tamaddon-Yalmeh, J., & Bahalgardi, B. (2017). Comparison of the effect of earmuffs and hocks on aggression of stone workers. *Bali Med. J., 6*(1), 164-168. <doi:https://doi.org/10.15562/bmj.v6i1.388> | Iran | 60 | Stone Workers | - Experimental study   - Comparison between experimental groups (earmuffs vs hocks)   - 1 month - Aggression Questionnaire   - Buss-Perry Aggression Questionnaire - Noise intensity   - CEL-450 Sound Level Meter - Blood pressure - Demographic data | - Psychological factors   - Aggression - Physiological factors   - Blood pressure | - Earplugs are more effective in reducing aggression in workers compared to earmuffs - Earplugs have a higher contribution in reducing factors of aggression (physical aggression, verbal aggression, anger and hostility) than earmuffs |
| Tavakolizadeh, J., Kianmehr, M., Tamaddon-Yalmeh, J., Bahalgardi, B., & Beheshti, M. H. (2019). Effect of Hearing Protection Devices on Anxiety and Depression of Stone Workers. *Journal of Research in Medical and Dental Science, 7*(3), 21-25. | Iran | 60 | Stone Workers | - Experimental study   - Comparison between experimental groups (earmuffs vs hocks)   - 1 month - Anxiety Questionnaire   - Hamilton Anxiety Scale - Depression Questionnaire   - Beck Depression Inventory - Noise intensity   - CEL-450 Sound Level Meter - Blood pressure - Demographic data | - Mental health   - Depression   - Anxiety - Physiological factors   - Blood pressure | - Anxiety decreased for all samples after using hearing protection devices - Anxiety decreased more for the earplugs group than the earmuffs group - No significant difference in depression before and after experimental period - Depression significantly decreased in earplugs group and increased in earmuffs group |
